# Lung cancer in never smokers in the UK Million Women Study

**DOI:** 10.1002/ijc.30084

**Published:** 2016-03-31

**Authors:** Kirstin Pirie, Richard Peto, Jane Green, Gillian K. Reeves, Valerie Beral, for the Million Women Study Collaborators

**Affiliations:** ^1^ Cancer Epidemiology Unit, Nuffield Department of Population Health, University of OxfordOxfordUnited Kingdom; ^2^ Clinical Trial Service Unit & Epidemiological Studies Unit (CTSU), Nuffield Department of Population Health, University of OxfordOxfordUnited Kingdom

**Keywords:** lung cancer, nonsmokers, women, prospective study

## Abstract

To assess directly the effects of various risk factors on lung cancer incidence among never smokers, large prospective studies are needed. In a cohort of 1.2 million UK women without prior cancer, half (634,039) reported that they had never smoked. Mean age at recruitment was 55 (SD5) years, and during 14 (SD3) years of follow‐up, 0.2% (1,469) of these never smokers developed lung cancer. Cox regression was used to estimate relative risks (RRs) of lung cancer for 34 potential risk factors, of which 31 were nonsignificant (*p* > 0.05). The remaining three risk factors were associated with a significantly increased incidence of lung cancer in never smokers: non‐white *vs*. white ethnicity (RR = 2.34, 95% CI 1.55–3.52, *p* < 0.001), asthma requiring treatment *vs*. not (RR = 1.32, 1.10–1.58, *p* = 0.003) and taller stature (height ≥ 165 cm *vs*. <160 cm: RR = 1.16, 1.03–1.32, *p* = 0.02). There was little association with other sociodemographic, anthropometric or hormonal factors, or with dietary intakes of meat, fish, fruit, vegetables and fiber. The findings were not materially affected by restricting the analyses to adenocarcinomas, the most common histological type among never smokers.

## Abstract 2

**What's new?**

What causes lung cancer in people who don't smoke? Because it's rare, researchers have a hard time teasing out exactly what factors contribute. Thanks to the Million Women Study, these authors were able to collect data from more than 634,000 women who had never smoked, of whom 1469 (0.2%) developed lung cancer. They analyzed 34 different characteristics, including lifestyle, medical, and socioeconomic factors, in relation to lung cancer status. Of the 34 risk factors, 3 showed a statistical connection with lung cancer: non‐white race, taller stature, and asthma requiring treatment.

Lung cancer is the leading cause of cancer death in the UK and worldwide.^1^ Smoking is much the most important cause of lung cancer in the UK,^2^ with disease rates reflecting both current and past smoking patterns. Hence, only a small proportion of all lung cancers arise in never smokers. Among never smokers, adenocarcinomas are the most common type, and squamous‐cell, small‐cell and large‐cell tumors are much less common.^3^, ^4^, ^5^, ^6^

Many possible risk factors for lung cancer other than smoking have been demonstrated or suggested, including some occupational exposures (*e.g*., to asbestos or silica dust), exposure to secondhand tobacco smoke, radon, indoor and outdoor air pollution, a history of prior lung disease, a family history of lung cancer, use of menopausal hormonal therapies, infection with human papillomavirus (HPV) and dietary factors such as high intakes of red or processed meat and low intakes of fruits and vegetables.^3^, ^4^, ^6^ Investigations of them are, however, susceptible to residual confounding by smoking, as many of these other factors are themselves associated with smoking. Hence, some prospective studies have tried to investigate the effects of other factors on lung cancer risk in nonsmokers, but as some excess lung cancer risk remains decades after stopping^7^ it is important to exclude not only current smokers but also ex‐smokers from such investigations. Moreover, prospective studies of never smokers need to be large to accrue sufficient numbers of cases to investigate reliably the risks associated with other exposures.

We report the relationships between various lifestyle and socioeconomic factors and lung cancer risk in a UK prospective study that included 634,039 middle‐aged women who reported at recruitment that they had never smoked. We selected 34 risk factors for investigation, all of which have been suggested previously to be associated with lung cancer risk, or are risk factors for other cancers.

## Material and Methods

Million Women Study methods are described elsewhere.^8^ In brief, participants were recruited into the Million Women Study in 1998, on average, through the National Health Service Breast Screening Programme, signing consent and completing a questionnaire about lifestyle, medical and socioeconomic factors. Women were resurveyed *via* postal questionnaires every 3–5 years. Study participants have a unique NHS number that links to the NHS Central Register. Dates of incident cancers, deaths and emigrations are routinely notified to us, with cancer site and histology coded to the International Classification of Diseases, ICD‐10 and ICD‐O.^9^, ^10^ The main outcomes of interest were incident lung cancer (ICD‐10 C34) and adenocarcinoma of the lung (the most common histological type in never smokers: ICD‐O codes 8140, 8211, 8250–8260, 8310, 8323, 8480–8490 or 8550). For analyses of exposure to secondhand smoke, small‐cell (ICD‐O 8041–8042), squamous‐cell (ICD‐O 8070–8072) and large‐cell (ICD‐O 8012) tumors were also investigated, as these are the types most strongly associated with smoking.

Women were asked about their smoking habits at recruitment and at each subsequent resurvey. Among the never smokers, we related the subsequent incidence of lung cancer to the following factors reported at recruitment: socioeconomic status (Townsend deprivation index^11^), highest educational qualification, age at menarche, duration of oral contraceptive use, number of full‐term pregnancies, age at first birth, height, body mass index, strenuous exercise, alcohol intake, diabetes requiring treatment, asthma requiring treatment, age at natural menopause or bilateral oophorectomy in postmenopausal never users of hormonal therapy for the menopause (HRT) and HRT use in postmenopausal women. HRT use was updated using information from the 3‐year survey and censored 4 years after last known use, to allow for changes in HRT use during the follow‐up period.

The following factors reported for the first time at the 3‐year survey were examined: birth weight, ethnic group (white, black, Asian, other), body size at age 10 years, either parent with a history of lung cancer, breast cancer, bowel cancer or diabetes, currently living with a partner, exposure to secondhand smoke at home as a child (when aged 10 or when born) or as an adult, currently in paid work, self‐rated health, hours of sleep per night, recent regular use of ibuprofen, aspirin or paracetamol and consumption of fruits and vegetables, fish, red or processed meats and estimated dietary fiber.

Ethics approval was provided by the Anglia and Oxford (now Cambridge South) Multicentre Research Ethics Committee, and all participants gave signed consent for follow‐up. Access to hospital admission data in England was approved by the Health and Social Care Information Centre.

### Statistical analysis

Women who reported at recruitment that they were smokers or ex‐smokers were excluded, as were women with any prior cancer (other than nonmelanoma skin cancer, ICD10 C44). Cox regression models (with attained age as the underlying time variable) yielded relative risks (RRs), adjusted where appropriate for age, geographical region (ten UK cancer registry regions), socioeconomic status (quintiles) and height (<160, 160–164, ≥165 cm). Missing values formed separate categories for height and socioeconomic status (<1.5% for each). For analyses of factors reported at recruitment, women contributed person‐years from the date of recruitment until registered with lung cancer, any other cancer (except nonmelanoma skin cancer), death or the end of follow‐up (January 1, 2014). Analyses were repeated just for responders to the 3‐year survey (with person‐years starting from that date) and then further restricted to exclude women who, despite reporting at recruitment that they had never smoked, reported at the 3‐year survey that they had.

To minimize the effects of early symptoms of lung cancer altering behavior, sensitivity analyses excluded the first 4 years of follow‐up. Plasma cotinine concentrations from 1,126 never smokers 9.0 (SD 1.4) years after recruitment were measured to check whether any were smoking, with levels >9.5 ng/mL considered indicative of active smoking.^12^

As ethnicity was reported only at the 3‐year survey, further information was sought from electronic hospital records *via* the national hospital episode statistics (HES).^13^ Using a nested case–control design, study lung cancer cases in England were matched to up to 20 controls on age, year of birth and region. Those with any HES record after recruitment and before lung cancer diagnosis that specified ethnicity were classified accordingly. Conditional logistic regression estimated lung cancer odds ratios for several ethnic minority groups relative to white ethnicity. For all analyses, 95% confidence intervals and two‐sided *p*‐values are presented, with no allowance for multiple comparisons. Calculations used Stata version 13.1.^14^

## Results

The Million Women Study recruited 1.2 million women without prior cancer and with information on smoking status, of whom 51% (634,039) reported that they had never smoked. There were marked differences in the distributions of many of the potential risk factors by smoking status. Compared to those who had ever smoked, never smokers lived in less deprived areas, were less likely to have been exposed to secondhand smoke at home (as a child or as an adult), had a later age at menopause, were less likely to use hormonal therapy for the menopause and had higher intakes of fruits and vegetables (Table 1).

**Table 1:** Characteristics of study participants at recruitment and at the 3‐year survey, by smoking status reported at recruitment

	Never smoked	Ever smoked
**Characteristics reported at recruitment**	*n* = 634,039	*n* = 607,726
Age (years)	56.3 (4.9)	55.9 (4.8)
Ever used oral contraceptives	56% (350,335)	64% (384,504)
Most deprived third of population	28% (172,827)	39% (236,759)
No educational qualifications	38% (233,208)	49% (290,512)
Asthma requiring treatment	7% (44,009)	9% (52,744)
Body mass index > 30 kg/m^2^	18% (106,072)	18% (103,808)
Strenuous exercise more than once a week	22% (133,334)	20% (117,692)
Menopause (natural or bilateral oophorectomy) at age <45 years	9% (19,778)	15% (27,358)
Postmenopausal^a^	72% (454,399)	71% (428,751)
Ever used HRT (in postmenopausal^1^ women)	47% (211,035)	52% (219,153)
>7 alcoholic drinks/week	14% (91,024)	25% (150,310)
**Follow‐up from recruitment**		
Person‐years (1000s)	8,886	8,250
Incident lung cancers	1,469	15,642
**Characteristics reported at 3‐year survey**	***n* = 422,009**	***n* = 354,238**
White ethnicity	98.7% (410,734)	99.4% (347,001)
In excellent/good health	80% (322,845)	72% (244,273)
Parent smoked at birth or age 10 of participant	81% (319,154)	88% (289,499)
Parent with lung cancer	11% (46,353)	12% (42,492)
Living with a partner	82% (342,217)	78% (271,049)
Partner smokes (in women living with a partner)	13% (42,940)	23% (61,591)
14+ pieces of fruit/week	39% (157,203)	32% (107,746)
4+ heaped tablespoons of vegetables/day	24% (97,551)	23% (78,153)
**Follow‐up from 3‐year survey**		
Person‐years (1000s)	6,090	5,015
Incident lung cancers	890	7,255

For all characteristics, entries are mean (and SD) or % (and numerator). Numbers do not always sum to totals due to missing values.

a Natural menopause, bilateral oophorectomy or aged ≥55 years at recruitment.

During 14 (SD 3) years of follow‐up, 0.2% (1,469/634,065) of the never smokers developed lung cancer at mean age 66 (SD 6) years. The lung cancer incidence rate in never smokers, standardized to the 2000 IARC world population for ages 50–79 years, was 14.3 per 100,000. Supporting Information Table 1 gives the age‐specific incidence rates in each 5‐year age group, which are similar to those in major US prospective studies, and subdivides them where possible by major histological categories. At all ages, the incidence rates were greater for adenocarcinomas than for small‐cell, squamous‐cell and large‐cell tumors combined. One‐third of all lung cancers in never smokers (456/1,469) were of unspecified or incompletely specified histology. Of the remaining 1,013 cases, adenocarcinoma was the most common histological type (682; 67%) followed by carcinoid (102; 10%), small‐cell (87; 9%), squamous‐cell (77; 8%) and large‐cell (15; 1%) tumors.

**Figure 1:**
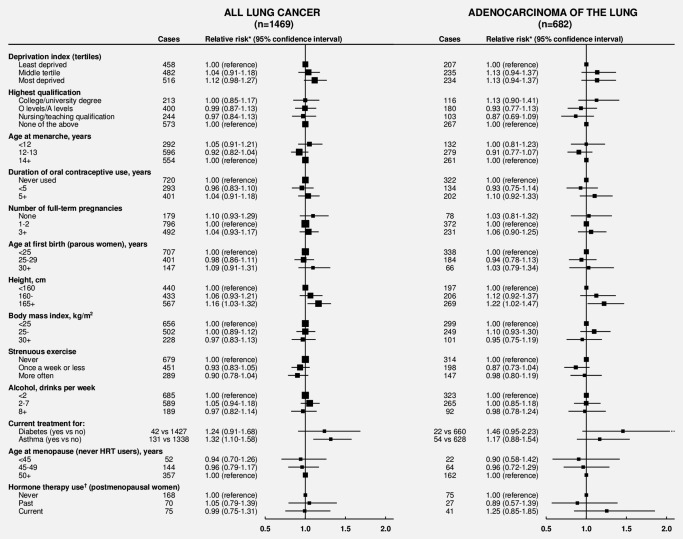
Never smokers: Relative risk* of incident lung cancer and adenocarcinoma of the lung by various factors. Numbers do not always sum to totals due to missing values. *N* = 1,469 lung cancers, of which there were 682 adenocarcinoma, 102 carcinoid, 87 small‐cell, 77 squamous‐cell, 15 large‐cell, 50 other specified types and 456 unspecified/nonspecific histological types. *Adjusted for age, region, deprivation quintile and height, where appropriate. †Hormone therapy use updated at the 3‐year survey and censored 4 years after last known use.

The relative risks for incident lung cancer, overall and separately for adenocarcinoma, associated with 14 factors reported at recruitment are shown in Figure 1, and associations with only two of these factors were statistically significant: lung cancer risk was greater for taller women, with a 16% increased risk for those at least 165 cm tall compared to those <160 cm tall (RR 1.16, 95% CI 1.03–1.32; *p* = 0.02), and for women with asthma requiring treatment (RR 1.32, 95% CI 1.10–1.58; *p* = 0.003). The association between asthma and lung cancer risk remained significant after excluding the first 4 years of follow‐up (RR 1.34, 95% CI 1.10–1.63; *p* = 0.003).

Postmenopausal women who were current users of hormone therapy for the menopause did not have an increased risk of lung cancer overall (RR 0.99, 95% CI 0.75–1.31). The relative risks for current estrogen only and current estrogen–progestagen use were 1.17 (95% CI 0.82–1.67) and 0.78 (95% CI 0.52–1.18), respectively, compared to never users (*p*_het_ = 0.1).

There was no significant association with deprivation, education, age at menarche, oral contraceptive use, number of full‐term pregnancies, age at first birth, body mass index, strenuous exercise, alcohol consumption, diabetes requiring treatment or menopausal age (*p* > 0.1 for each). The associations for adenocarcinoma were similar to those for all lung cancer (Fig. 1).

Of the never smokers at recruitment, 68% (431,173) completed another study questionnaire 3.3 (SD1.1) years later, of whom 422,009 then had no prior cancer. All relationships shown in Figure 1 were similar when analyses were restricted to responders to the 3‐year survey, and after excluding from this date onward the 5% (20,515/422,009) of never smokers at recruitment who reported at the 3‐year survey that they had ever smoked (Supporting Information Fig. 1). Of these 20,515 who had in fact smoked, 99% (20,287) were ex‐smokers who had on average stopped decades earlier [at mean age 26 (SD 8) years] and had smoked only lightly [5 (SD 4) cigarettes per day]. These 20,515 women had a slightly elevated risk of lung cancer compared to the women who on both questionnaires reported never having smoked (RR 1.36, 95% CI 1.04–1.77; *p* = 0.03), and were excluded from subsequent analyses. Few of the never smokers at recruitment were active smokers during follow‐up; of those replying 3 years later only 0.05% (228/422,016) reported current smoking, and of those who had cotinine measured around 9 years after recruitment only 0.3% (3/1126) had values suggestive of active smoking.

**Figure 2:**
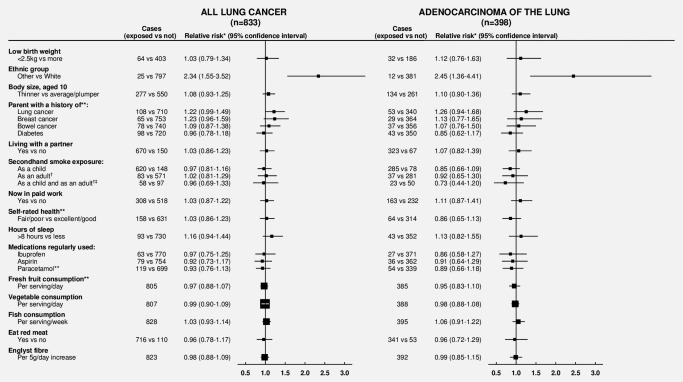
Never smokers: Relative risk* of incident lung cancer and adenocarcinoma of the lung by various factors reported about 3 years after recruitment. Numbers do not always sum to totals due to missing values. *N* = 833 lung cancers, of which there were 398 adenocarcinoma, 66 carcinoid, 37 small‐cell, 46 squamous‐cell, five large‐cell, 33 other specified types and 248 unspecified/nonspecific histological types. *Adjusted for age, region, deprivation quintile and height. **Question not included on 2% of questionnaires. †Restricted to those who lived with a partner (and therefore at risk of exposure to secondhand smoke through living with a partner who smokes). ‡Reference group are those with neither exposure.

Figure 2 shows the relationships between 20 other factors reported for the first time at the 3‐year survey and lung cancer risk, both overall and just for adenocarcinomas. Women who reported their ethnicity not to be white (*i.e*., black, Asian or other non‐white) had an increased risk of lung cancer (RR 2.34, 95% CI 1.55–3.52; *p* < 0.001) compared to those who reported themselves to be white. Since our questionnaire information on ethnicity was limited, we sought further information for study participants in England from hospital admission records with specified ethnicity, and a nested case–control analysis was carried out for women with such a record. Based on this analysis of 953 cases and 28,488 matched controls, the odds ratios and numbers of cases/controls for women of black, South Asian and Chinese ethnicity were 1.73 (95% CI 0.98–3.04; 13/229), 1.04 (0.58–1.86; 12/349) and 2.70 (1.08–6.76; 5/56), respectively, compared to women of white ethnicity (912/27,447).

In never smokers, there was no significant association between lung cancer risk and reported exposure to secondhand smoke at home, either as a child or through living with a partner who smoked. The three types of lung cancer most strongly associated with smoking (*i.e*., with the greatest smoker *vs*. nonsmoker RR) are small‐cell, squamous‐cell and large‐cell tumors, but in aggregate there were only 69 such cancers among women living with a partner of known smoking habits (Supporting Information Fig. 2). Among women living with a partner, secondhand smoke exposure at home both as a child and as an adult was not associated with a greater risk of lung cancer than not being exposed at either time, either overall (RR 0.96, 95% CI 0.69–1.33; 58 *vs*. 97 cases), for adenocarcinomas (RR 0.73, 95% CI 0.44–1.20; 23 *vs*. 50 cases) or for small‐cell, squamous‐cell and large‐cell tumors combined (RR 1.48, 95% CI 0.79–2.76; 10 *vs*. 10 cases) (Fig. 2, Supporting Information Fig. 2).

The relative risk of lung cancer associated with having a mother or father with a history of lung cancer was not conventionally significant (RR 1.22, 95% CI 0.99–1.49, *p* = 0.06). Among nonsmoking women who reported that a parent had had lung cancer, 91% (38,405/42,208) also reported that their affected parent had smoked either when the woman was born or when she was 10 years old.

There were no significant associations of lung cancer risk with dietary intakes of fruit, vegetables, meat, fish or fiber, with sleep duration, with regular use of aspirin, ibuprofen or paracetamol or with any of the other factors examined (Fig. 2).

## Discussion

In this prospective study of 1.2 million women, half (0.6 million) reported at recruitment that they had never smoked, and during 14 years of follow‐up 0.2% (1469) of these never smokers developed lung cancer. Of 34 risk factors analyzed, 31 were not significantly related to risk. However, lung cancer incidence was somewhat higher for women of non‐white ethnicity, of taller stature and for women with asthma requiring treatment. Consistent with findings of previous studies,^3^, ^4^, ^5^, ^6^ adenocarcinomas accounted for two‐thirds of the nonsmoker lung cancers with specified histology, and when restricted to adenocarcinomas the findings were essentially unchanged.

The age‐standardized incidence rate of lung cancer among never smokers in the present prospective study is comparable with the rates reported in other such studies (from 14.4 to 20.8 per 100,000 aged 40–79)^5^ and in a pooled analysis (12.4 per 100,000 women of European descent aged 40–84).^15^ With 1,469 incident cases, however, this is the largest prospective study to date of risk factors for lung cancer in women who have never smoked. Other prospective studies that have reported on risk factors for lung cancer in never smokers have typically included fewer than 200 cases, with the largest studies to date being NIH‐AARP^16^ and the Shanghai Women's Health study,^17^ each of which included about 500 incident lung cancers in never smokers.

Smoking status was self‐reported, and among those recruited as never smokers there was some misclassification, particularly of ex‐smokers who had stopped some decades previously. Of the never smokers at recruitment who completed the 3‐year survey, 5% reported at the later survey that they had in fact smoked. On average, these women were ex‐smokers who had stopped at age 26 years and had smoked five cigarettes per day, but even with these low levels of exposure they still had a slight excess risk of lung cancer. Excluding these women did not materially affect any of the risk estimates and about 99% of the 1469 analysed lung cancer cases were not caused by active smoking. (Supporting Information Fig. 1). Few who reported at recruitment that they were never smokers would have smoked during the study, as cotinine measurements on over 1,000 women about 9 years later found only 0.3% had cotinine levels suggestive of current smoking.

The published evidence on risk factors for lung cancer other than smoking has been reviewed elsewhere,^3^, ^4^, ^6^ and most associations are inconsistent across studies. When looking at factors other than active smoking, it is important to exclude as far as possible both current smokers and ex‐smokers, as adjusting for smoking can produce misleading results due to residual confounding; moreover, the associations for other risk factors may differ between smokers and never smokers. In populations of European origin, however, never‐smoker lung cancer rates are so low that restriction of analyses to never smokers means that few prospective studies have sufficient numbers for analysis.

There are, however, some Asian populations where few women smoke but female lung cancer rates are high,^15^, ^18^ and in them other factors, such as exposure to indoor coal smoke emissions, have been associated with lung cancer risk.^3^, ^19^ Nonsmoking women of Asian and African‐American descent living in the USA have also been reported to have greater lung cancer mortality than women of European descent.^15^ We found a significantly increased incidence of lung cancer among women who reported non‐white *vs*. white ethnicity (RR = 2.3), but this was based on only small numbers of cases and the information on ethnicity from our questionnaire was limited. A nested case–control analysis of ethnicity based on electronic hospital records also found evidence of an increased risk of lung cancer for women of Chinese (OR = 2.7) and black (OR = 1.7) ethnicity compared to women of white ethnicity, but numbers were insufficient to estimate risks reliably.

Previous studies of exposure to secondhand smoke and lung cancer risk have been reviewed extensively,^20^, ^21^, ^22^ and the most recent monograph from the International Agency for Research in Cancer (IARC) concludes that there is sufficient evidence that secondhand smoke causes some risk of lung cancer.^20^ Assessing an individual's true exposure to secondhand smoke is, however, difficult, and in this and other studies some misclassification of exposure will have occurred. A limitation of this study is that we assessed household exposure only by the presence of a smoking parent or partner, and we did not seek information on sources of exposure outside the home or on exposure duration. The strengths of this study, however, are that it was prospective, has large numbers of incident cases of primary lung cancer and could exclude smokers and ex‐smokers reasonably reliably from our analyses. This is important because some excess risk of lung cancer remains long after stopping smoking,^7^ and ex‐smokers could well be more likely to have been exposed to secondhand smoke than never smokers,^23^ *e.g*., through living with other smokers. Hence, in studies that deliberately or inadvertently include enough ex‐smokers to account for an appreciable proportion of the lung cancers, residual confounding could lead to some of the excess hazard among them from prior active smoking being wrongly attributed to secondhand smoke. This may be why this study suggests less effect of secondhand smoke on the overall risk of lung cancer than previous studies did. If, however, attention is restricted to small‐cell, squamous‐cell and large‐cell tumors (as these are much more closely related to active smoking than any other histological type of lung cancer), the relative risk for exposure to secondhand smoke at home both as a child and as an adult is increased, but with wide confidence limits.

It has been suggested that various chronic lung diseases, including asthma, could increase lung cancer risk, perhaps through damage caused by inflammation or trauma.^24^, ^25^ A pooled analysis of five published studies in never smokers^24^ found a nonsignificant relative risk of 1.17 for lung cancer in those with asthma compared to those without, which is consistent with the relative risk of 1.32 (95% CI 1.10–1.58) reported here. The association in our study appeared to persist after excluding the first 4 years of follow‐up to avoid early lung cancer symptoms being misdiagnosed as asthma. Even if asthma does increase the nonsmoker lung cancer risk by a third, this would translate to only a small absolute excess risk of about 0.1% by age 80.

We found no significant difference in lung cancer risk between postmenopausal never smokers who used hormone therapy for the menopause and those who did not. Results from other prospective studies are mixed^26^, ^27^, ^28^, ^29^, ^30^, ^31^, ^32^, ^33^, ^34^; of those that reported results in never smokers, one reported a decreased risk,^33^ two reported a nonsignificantly increased risk^32^, ^34^ and others reported no association.^27^, ^30^, ^31^

In earlier analyses of this cohort that included only 667 lung cancers in never smokers, a 15% increased risk per 10 cm increase in height was reported.^35^ With longer follow‐up and 1,469 cases the findings remain similar, suggesting taller women have a slightly higher risk of lung cancer. Being tall is associated with increased risks for most types of cancer.^35^ The mechanisms remain unclear, but may involve increased cell turnover as a result of having more cells or increased growth factor levels.^35^ Again, however, the absolute excess risk by age 80 is small, differing by less than 0.1% between the tallest and shortest groups. We found no significant associations of risk with other anthropometric factors, including birth weight, body size at age 10 and body mass index.

We did not collect information on radon exposure, indoor or outdoor air pollution or occupational exposures, which have all been implicated as risk factors for lung cancer.^3^, ^4^, ^6^ However, occupational exposure to factors such as asbestos, silica or arsenic is much less common in women than in men,^36^, ^37^, ^38^ and factors such as radon and asbestos are of much less absolute importance in never smokers than in smokers.^5^, ^20^, ^36^ Analyses were stratified by region, which would partially account for the geographical variation in residential radon levels that occur across the UK.

In this large cohort of UK women, only three of the 34 factors examined were associated with significantly increased lung cancer risk in never smokers: non‐white ethnicity, tall stature and asthma requiring treatment, but none carried a large absolute risk. In contrast with the small risks in nonsmokers due to factors other than smoking, in this population current smoking produces a relative risk of about 20 for lung cancer mortality, and smoking cessation can greatly reduce this large absolute hazard.^7^

## Supporting information

Supporting Information

## References

[1] Ferlay J , Soerjomataram I , Ervik M , et al. GLOBOCAN 2012 v1.0, cancer incidence and mortality worldwide: IARC CancerBase No. 11. 2013 Available at: http://globocan.iarc.fr (accessed October 3, 2014).

[2] Parkin DM , Boyd L , Walker LC. The fraction of cancer attributable to lifestyle and environmental factors in the UK in 2010. Br J Cancer 2011;105:S77–S81. 22158327 10.1038/bjc.2011.489 PMC3252065

[3] Sun S , Schiller JH , Gazdar AF. Lung cancer in never smokers—a different disease. Nat Rev Cancer 2007;7:778–90. 17882278 10.1038/nrc2190

[4] Samet JM , Avila‐Tang E , Boffetta P , et al. Lung cancer in never smokers: clinical epidemiology and environmental risk factors. Clin Cancer Res 2009;15:5626–45. 19755391 10.1158/1078-0432.CCR-09-0376 PMC3170525

[5] Wakelee HA , Chang ET , Gomez SL , et al. Lung cancer incidence in never smokers. J Clin Oncol 2007;25:472–8. 17290054 10.1200/JCO.2006.07.2983 PMC2764546

[6] Subramanian J , Govindan R. Lung cancer in never smokers: a review. J Clin Oncol 2007;25:561–70. 17290066 10.1200/JCO.2006.06.8015

[7] Pirie K , Peto R , Reeves GK , et al. The 21^st^ century hazards of smoking and benefits of stopping: a prospective study of one million women in the UK. Lancet 2013;381:133–41. 23107252 10.1016/S0140-6736(12)61720-6 PMC3547248

[8] The Million Women Study Collaborative Group. The Million Women Study: design and characteristics of the study population. Breast Cancer Res 1999;1:73–80. 11056681 10.1186/bcr16 PMC13913

[9] World Health Organization. International statistical classification of diseases and related health problems, 10th revision. Geneva: World Health Organization, 1992.

[10] Fritz A , Percy C , Jack A. International classification of diseases for oncology, 3rd edn. Geneva: World Health Organization, 2000.

[11] Townsend P , Phillimore P , Beattie A. Health and deprivation: inequality and the north. London: Croon Helm, 1988.

[12] Jarvis MJ , Fidler J , Mindell J , et al. Assessing smoking status in children, adolescents and adults: cotinine cut‐points revisited. Addiction 2008;103:1553–61. 18783507 10.1111/j.1360-0443.2008.02297.x

[13] Hospital Episode Statistics . Available at: http://www.hscic.gov.uk/hes (accessed June 8, 2014).

[14] StataCorp . Stata statistical software: release 13. College Station, TX: StataCorp LP, 2013.

[15] Thun MJ , Hannan LM , Adams‐Campbell LL , et al. Lung cancer occurrence in never‐smokers: an analysis of 13 cohorts and 22 cancer registry studies. PLoS Med 2008;5(9):e185. 10.1371/journal.pmed.0050185 PMC2531137 18788891

[16] Lam TK , Moore SC , Brinton LA , et al. Anthropometric measures and physical activity and the risk of lung cancer in never‐smokers: a prospective cohort study. PLoS One 2013;8(8):e70672. 10.1371/journal.pone.0070672 PMC3734257 23940620

[17] Yang WS , Yang Y , Yang G , et al. Pre‐existing type 2 diabetes and risk of lung cancer: a report from two prospective cohort studies of 133 024 Chinese adults in urban Shanghai. BMJ Open 2014;4:e004875. 10.1136/bmjopen-2014-004875 PMC4091264 24993754

[18] Lee PN , Forey BA. Indirectly estimated absolute lung cancer mortality rates by smoking status and histological type based on a systematic review. BMC Cancer 2013;13:189. 10.1186/1471-2407-13-189 PMC3639928 23570286

[19] Sisti J , Boffetta P. What proportion of lung cancer in never‐smokers can be attributed to known risk factors? Int J Cancer 2012;131:265–75. 22322343 10.1002/ijc.27477 PMC3359408

[20] International Agency for Research on Cancer. IARC monographs on the evaluation of carcinogenic risks to humans, vol. 100E: A review of human carcinogens: personal habits and indoor combustions. Lyon: IARC, 2012. PMC4781577 23193840

[21] U.S. Department of Health and Human Services . The health consequences of smoking: a report of the surgeon general. Washington, DC: US Dept of Health and Human Services, Centers for Disease Control and Prevention, National Center for Chronic Disease Prevention and Health Promotion, Office on Smoking and Health, 2004.

[22] International Agency for Research on Cancer. Monographs on the evaluation of carcinogenic risks to humans, vol. 83: Tobacco smoke and involuntary smoking. Lyon: IARC, 2004. PMC4781536 15285078

[23] U.S. Department of Health and Human Services . The health consequences of involuntary exposure to tobacco smoke: a report of the surgeon general. Atlanta, GA: US Dept of Health and Human Services, Centers for Disease Control and Prevention, National Center for Chronic Disease Prevention and Health Promotion, Office on Smoking and Health, 2006.

[24] Rosenberger A , Bickeböller H , McCormack V , et al. Asthma and lung cancer risk: a systematic investigation by the international lung cancer consortium. Carcinogenesis 2012;33:587–97. 22198214 10.1093/carcin/bgr307 PMC3291861

[25] Brenner DR , Boffetta P , Duell EJ , et al. Previous lung diseases and lung cancer risk: a pooled analysis from the international lung cancer consortium. Am J Epidemiol 2012;176:573–85. 22986146 10.1093/aje/kws151 PMC3530374

[26] Chlebowski RT , Schwartz AG , Wakelee H , et al. Oestrogen plus progestin and lung cancer in postmenopausal women (Women's Health Initiative trial): a post‐hoc analysis of a randomised controlled trial. Lancet 2009;374:1243–51. 19767090 10.1016/S0140-6736(09)61526-9 PMC2995490

[27] Baik CS , Strauss GM , Speizer FE , et al. Reproductive factors, hormone use, and risk for lung cancer in postmenopausal women, the nurses' health study. Cancer Epidemiol Biomark Prev 2010;19:2525–33. 10.1158/1055-9965.EPI-10-0450 PMC2952036 20739629

[28] Slatore CG , Chien JW , Au DH , et al. Lung cancer and hormone replacement therapy: association in the vitamins and lifestyle study. J Clin Oncol 2010;28:1540–6. 20159813 10.1200/JCO.2009.25.9739 PMC2849773

[29] Brinton LA , Gierach GL , Andaya A , et al. Reproductive and hormonal factors and lung cancer risk in the NIH‐AARP diet and health study cohort. Cancer Epidemiol Biomarkers Prev 2011;20:900–11. 21467241 10.1158/1055-9965.EPI-10-1325 PMC3507989

[30] Kabat GC , Miller AB , Rohan TE. Reproductive and hormonal factors and risk of lung cancer in women: a prospective cohort study. Int J Cancer 2007;120:2214–20. 17278095 10.1002/ijc.22543

[31] Clague J , Reynolds P , Sullivan‐Halley J , et al. Menopausal hormone therapy does not influence lung cancer risk: results from the California Teachers Study. Cancer Epidemiol Biomarkers Prev 2011;20:560–4. 21266521 10.1158/1055-9965.EPI-10-1182 PMC3065239

[32] Liu Y , Inoue M , Sobue T , et al. Reproductive factors, hormone use and the risk of lung cancer among middle‐aged never‐smoking Japanese women: a large‐scale population‐based cohort study. Int J Cancer 2005;117:662–6. 15929081 10.1002/ijc.21229

[33] Rodriguez C , Feigelson HS , Deka A , et al. Postmenopausal hormone therapy and lung cancer risk in the Cancer Prevention Study II Nutrition Cohort. Cancer Epidemiol Biomarkers Prev 2008;17:655–60. 18349283 10.1158/1055-9965.EPI-07-2683

[34] Chlebowski RT , Anderson GL , Manson JE , et al. Lung cancer among postmenopausal women treated with estrogen alone in the Women's Health Initiative Randomized Trial. J Natl Cancer Inst 2010;102:1413–21. 20709992 10.1093/jnci/djq285 PMC2943522

[35] Green J , Cairns BJ , Casabonne D , et al. Height and cancer incidence in the Million Women Study: prospective cohort, and meta‐analysis of prospective studies of height and total cancer risk. Lancet Oncol 2011;12:785–94. 21782509 10.1016/S1470-2045(11)70154-1 PMC3148429

[36] Clément‐Duchêne C , Vignaud JM , Stoufflet A , et al. Characteristics of never smoker lung cancer including environmental and occupational risk factors. Lung Cancer 2010;67:144–50. 19464070 10.1016/j.lungcan.2009.04.005

[37] Pohlabeln H , Boffetta P , Ahrens W , et al. Occupational risks for lung cancer among nonsmokers. Epidemiology 2000;11:532–8. 10955405 10.1097/00001648-200009000-00008

[38] Couraud S , Souquet PJ , Paris C , et al. BioCAST/IFCT‐1002: epidemiological and molecular features of lung cancer in never‐smokers. Eur Respir J 2015;45:1403–14. 25657019 10.1183/09031936.00097214

