# Supplementary material for: Lung cancer in never smokers in the UK Million Women Study
Source: Int J Cancer. 2016 Mar 31;139(2):347–54. doi: 10.1002/ijc.30084 (PMC4864444; doi:10.1002/ijc.30084)
Supplement: Supplementary file 1 — Supporting Information [file IJC-139-347-s001.pdf]

Web Table 1: Annual lung cancer incidence rates per 100,000 prospectively observed UK female never-smokers, by age at risk

		All lung cancer		Adenocarcinoma		Small-cell, squamous-cell and large-cell		Other specified type*	
Age at risk	Person-years	Cases	Rate**	Cases	Rate	Cases	Rate	Cases	Rate
50-54	758,866	39	5.1	22	2.9	4	0.5	4	0.5
55-59	1,746,857	115	6.6	50	2.9	16	0.9	7	0.4
60-64	2,499,932	340	13.6	161	6.4	44	1.8	40	1.6
65-69	2,170,829	425	19.6	204	9.4	47	2.2	45	2.1
70-74	1,212,951	343	28.3	153	12.6	48	4.0	39	3.2
75-79	438,427	178	40.6	83	18.9	17	3.9	15	3.4
80-84	57,690	29	50.3	9	15.6	3	5.2	2	3.5

* Mainly carcinoid (ICD-O 8240/3) or neuroendocrine (ICD-O 8246/3) tumours. Of the total of 1469 lung cancers, only 1013 were of a specified type; of the remaining 456 tumours, half were completely unspecified and half were specified incompletely (ie, specified only as not being small cell cancers; ICD-O 8046/3).

** The 6 age-specific lung cancer incidence rates at ages 50-79 are remarkably similar to the lung cancer mortality rates at these ages observed prospectively among US female never-smokers, which were 5, 8, 14, 18, 28, and 42 per 100,000 (Lancet 1992;339:1268-78).

Web Figure 1. Never smokers at recruitment: Relative risk* of incident lung cancer by various factors; (A) restricted to 422,009 responders to the 3-year survey, and (B) excluding 20,515 women who reported at the 3-year survey that they had ever smoked.

	(A) 3-YEAR SURVEY RESPONDERS (n=890 cases)		(B) EXCLUDING WOMEN WHO REPORTED EVER HAVING SMOKED AT THE 3-YEAR SURVEY (n=833 cases)	
	Cases	Relative risk* (95% confidence interval)	Cases	Relative risk* (95% confidence interval)
**Deprivation index (tertiles)**				
Least deprived	286	1.00 (reference)	268	1.00 (reference)
Middle tertile	313	1.11 (0.95-1.31)	298	1.13 (0.96-1.33)
Most deprived	282	1.13 (0.96-1.33)	260	1.11 (0.94-1.32)
**Highest qualification**				
College/university degree	138	0.93 (0.76-1.14)	124	0.88 (0.71-1.10)
O levels/A levels	279	1.06 (0.89-1.25)	264	1.04 (0.88-1.24)
Nursing/teaching qualification	152	0.99 (0.81-1.20)	141	0.96 (0.78-1.18)
None of the above	308	1.00 (reference)	293	1.00 (reference)
**Age at menarche, years**				
<12	196	1.19 (1.00-1.43)	188	1.21 (1.01-1.46)
12-13	372	0.97 (0.84-1.13)	342	0.94 (0.81-1.10)
14+	312	1.00 (reference)	294	1.00 (reference)
**Duration of oral contraceptive use, years**				
Never used	425	1.00 (reference)	402	1.00 (reference)
<5	185	0.94 (0.79-1.13)	170	0.93 (0.77-1.12)
5+	256	1.04 (0.89-1.23)	239	1.04 (0.88-1.23)
**Number of full-term pregnancies**				
None	110	1.09 (0.89-1.34)	104	1.10 (0.89-1.37)
1-2	491	1.00 (reference)	458	1.00 (reference)
3+	288	1.03 (0.89-1.20)	270	1.04 (0.89-1.21)
**Age at first birth (parous women), years**				
<25	408	1.00 (reference)	385	1.00 (reference)
25-29	262	1.03 (0.88-1.20)	244	1.01 (0.86-1.19)
30+	90	1.08 (0.86-1.36)	81	1.04 (0.81-1.32)
**Height, cm**				
<160	262	1.00 (reference)	246	1.00 (reference)
160-	261	1.02 (0.86-1.21)	245	1.02 (0.85-1.22)
165+	358	1.13 (0.96-1.33)	334	1.13 (0.95-1.33)
**Body mass index, kg/m^2**				
<25	426	1.00 (reference)	398	1.00 (reference)
25-	299	0.98 (0.85-1.14)	285	1.00 (0.86-1.17)
30+	127	0.96 (0.79-1.17)	116	0.95 (0.77-1.17)
**Strenuous exercise**				
Never	379	1.00 (reference)	362	1.00 (reference)
Once a week or less	293	0.94 (0.81-1.10)	270	0.91 (0.77-1.07)
More often	191	0.93 (0.78-1.10)	178	0.90 (0.76-1.08)
**Alcohol, drinks per week**				
<2	390	1.00 (reference)	374	1.00 (reference)
2-7	384	1.12 (0.97-1.29)	356	1.10 (0.95-1.27)
8+	116	0.95 (0.77-1.17)	103	0.91 (0.73-1.13)
**Current treatment for:**				
Diabetes (yes vs no)	23 vs 867	1.36 (0.90-2.06)	21 vs 812	1.33 (0.86-2.05)
Asthma (yes vs no)	79 vs 811	1.36 (1.08-1.72)	74 vs 759	1.37 (1.08-1.74)
**Age at menopause (never HRT users), years**				
<45	25	0.78 (0.51-1.18)	25	0.83 (0.55-1.26)
45-49	84	0.89 (0.69-1.15)	83	0.93 (0.72-1.21)
50+	233	1.00 (reference)	218	1.00 (reference)
**Hormone therapy use† (postmenopausal women)**				
Never	88	1.00 (reference)	80	1.00 (reference)
Past	42	0.96 (0.66-1.39)	41	1.04 (0.67-1.61)
Current	38	1.05 (0.71-1.55)	33	1.00 (0.61-1.65)

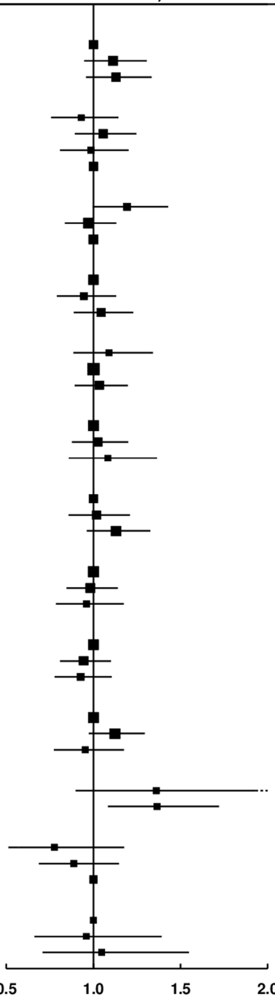

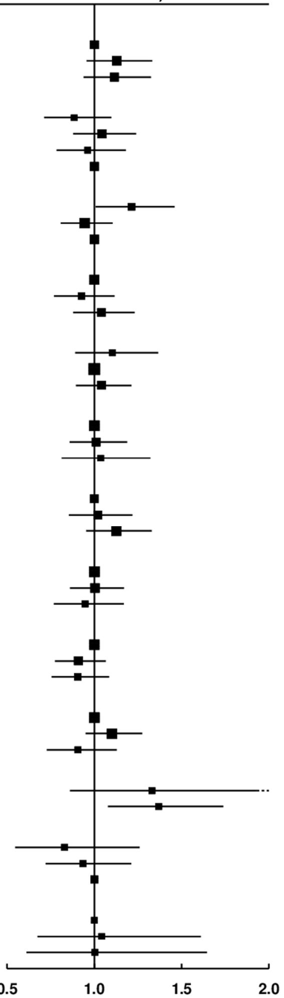

Numbers do not always add up to totals due to missing values. *Adjusted for age, region, deprivation quintile and height, where appropriate. †Updated at the 3-year survey and censored 4 years after last known use.

There were 890 cases among women who responded to the 3-year survey and 646 in those who did not. Of the 890, 57 (890-833) were among the women who were excluded from all later analyses as they reported at the 3-year survey that they had in fact smoked, mostly decades previously (and had RR 1.36, suggesting 15 of these 57 lung cancers were due to smoking).

This suggests that of the 1469 cases kept in our main analyses, 0.7% (646 x 15/890 = 11 cases) were caused by previous smoking that could have been revealed by the 3-year questionnaire.

Web Figure 2. Never smokers at recruitment and at the 3-year survey: Relative risk* of incident adenocarcinoma and small-cell, squamous-cell and large-cell tumours of the lung by exposure to secondhand tobacco smoke.

ADENOCARCINOMA

	Cases (exposed vs not)	Relative risk* (95% confidence interval)
**Living with a partner**		
Yes vs no	323 vs 67	1.07 (0.82-1.39)
**Secondhand smoke exposure:**		
As a child	285 vs 78	0.85 (0.66-1.09)
As an adult†	37 vs 281	0.92 (0.65-1.30)
As a child and as an adult†‡	23 vs 50	0.73 (0.44-1.20)

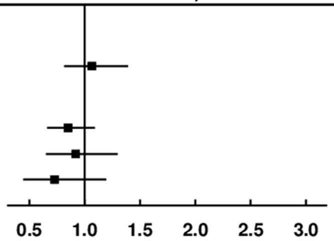

SMALL-CELL, SQUAMOUS-CELL AND LARGE-CELL

	Cases (exposed vs not)	Relative risk* (95% confidence interval)
**Living with a partner**		
Yes vs no	71 vs 16	1.11 (0.64-1.93)
**Secondhand smoke exposure:**		
As a child	69 vs 13	1.15 (0.64-2.09)
As an adult†	12 vs 57	1.45 (0.77-2.73)
As a child and as an adult†‡	10 vs 10	1.48 (0.79-2.76)

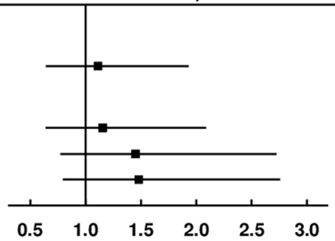

*Adjusted for age, region, deprivation quintile and height.

†Restricted to those who lived with a partner (and therefore at risk of exposure to secondhand smoke through living with a partner who smokes). ‡Reference group are those with neither exposure.
